# A Bedside Compression Test to Differentiate Pulsatile Varicose Veins

**DOI:** 10.1002/ccr3.72331

**Published:** 2026-03-19

**Authors:** Manabu Ida, Takeshi Ueda

**Affiliations:** ^1^ Department of Emergency and General Medicine Rakuwakai Marutamachi Hospital Kyoto Japan

**Keywords:** bedside compression test, physical examination, pulsatile varicose veins, systemic venous hypertension

## Abstract

A simple bedside compression test was performed in a patient with pulsatile varicose veins. Venous pulsation persisted during femoral arterial compression but disappeared during femoral venous compression, supporting venous pulsatility secondary to systemic venous hypertension.

## Key Clinical Message

A simple bedside compression test can help differentiate venous pulsatility caused by systemic venous hypertension from arterial inflow in patients with pulsatile varicose veins, thereby supporting appropriate clinical management.

## 1 Question

A woman in her nineties with right‐sided heart failure presented with pulsatile varicose veins in her lower extremities. How can these be differentiated at the bedside, and how does this distinction affect management?

## 2 Answer

**FIGURE 1:**
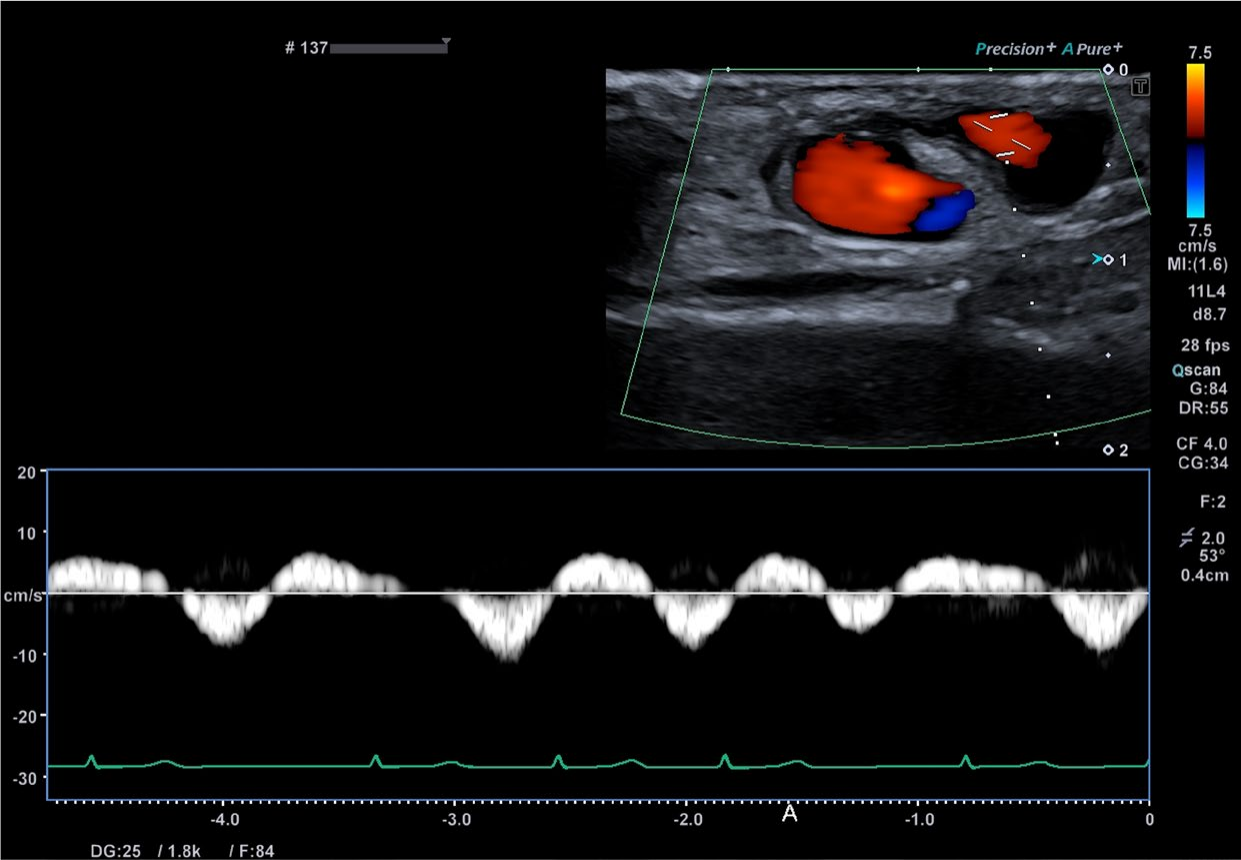
Pulsed‐wave Doppler ultrasonography at baseline. Baseline pulsed‐wave Doppler shows a low‐velocity, bidirectional venous waveform synchronized with the cardiac cycle.

**FIGURE 2:**
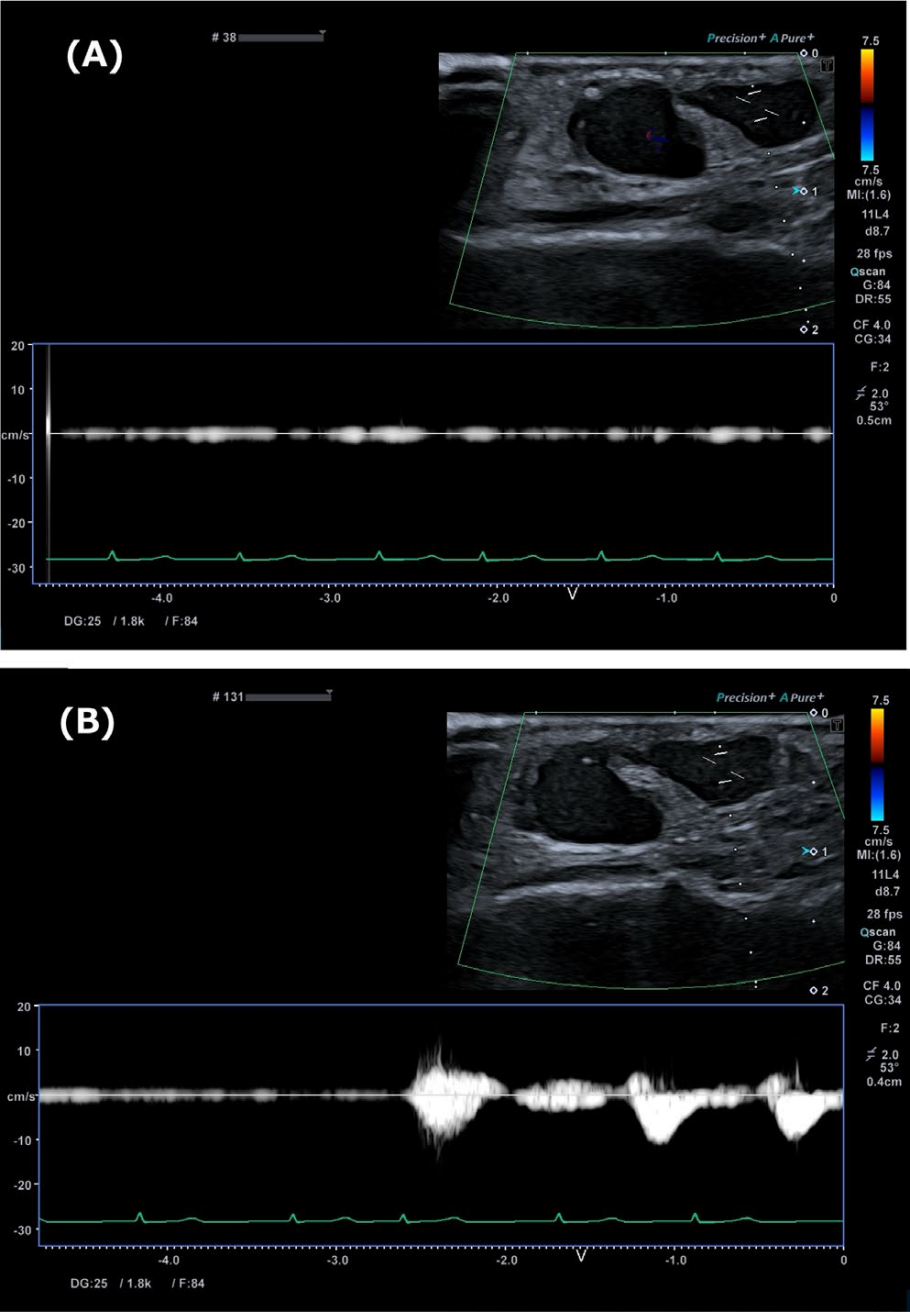
Pulsed‐wave Doppler ultrasonography during compression testing. (A) Femoral vein compression abolishes pulsatile venous flow. (B) After switching from venous to femoral artery compression, venous pulsatility reappears and persists, indicating venous pressure transmission rather than arterial inflow.

Sequential compression of the femoral artery and vein helps distinguish the hemodynamic origin of pulsatility (Video S1). This response reflects venous pressure transmission rather than arterial inflow or arteriovenous communication. Doppler ultrasonography confirmed venous pulsatility (Figures 1 and 2). This maneuver is a screening tool and does not replace definitive imaging. Recognizing the hemodynamic origin guides appropriate management toward cardiac rather than local treatment [1, 2].

## Author Contributions

**Manabu Ida:** conceptualization, investigation, methodology, visualization, writing – original draft, writing – review and editing. **Takeshi Ueda:** supervision, writing – review and editing.

## Funding

The authors have nothing to report.

## Consent

Written informed consent was obtained from the patient for the publication of this case and accompanying images and videos.

## Conflicts of Interest

The authors declare no conflicts of interest.

## Supporting information


**Video S1:** Bedside compression test for pulsatile varicose veins. The patient was examined in the supine position. Manual compression was sequentially applied to the femoral artery and then the femoral vein for several seconds each, while observing changes in venous pulsatility. Venous pulsation persisted during arterial compression but disappeared during subsequent venous compression.

**Data S1:** Supporting Information.

## Data Availability

Data sharing is not applicable to this article as no datasets were generated or analysed during the current study.
