# Supplementary figures and images for: A Bedside Compression Test to Differentiate Pulsatile Varicose Veins
Source: Clin Case Rep. 2026 Mar 19;14(3):e72331. doi: 10.1002/ccr3.72331 (PMC13093701; doi:10.1002/ccr3.72331)

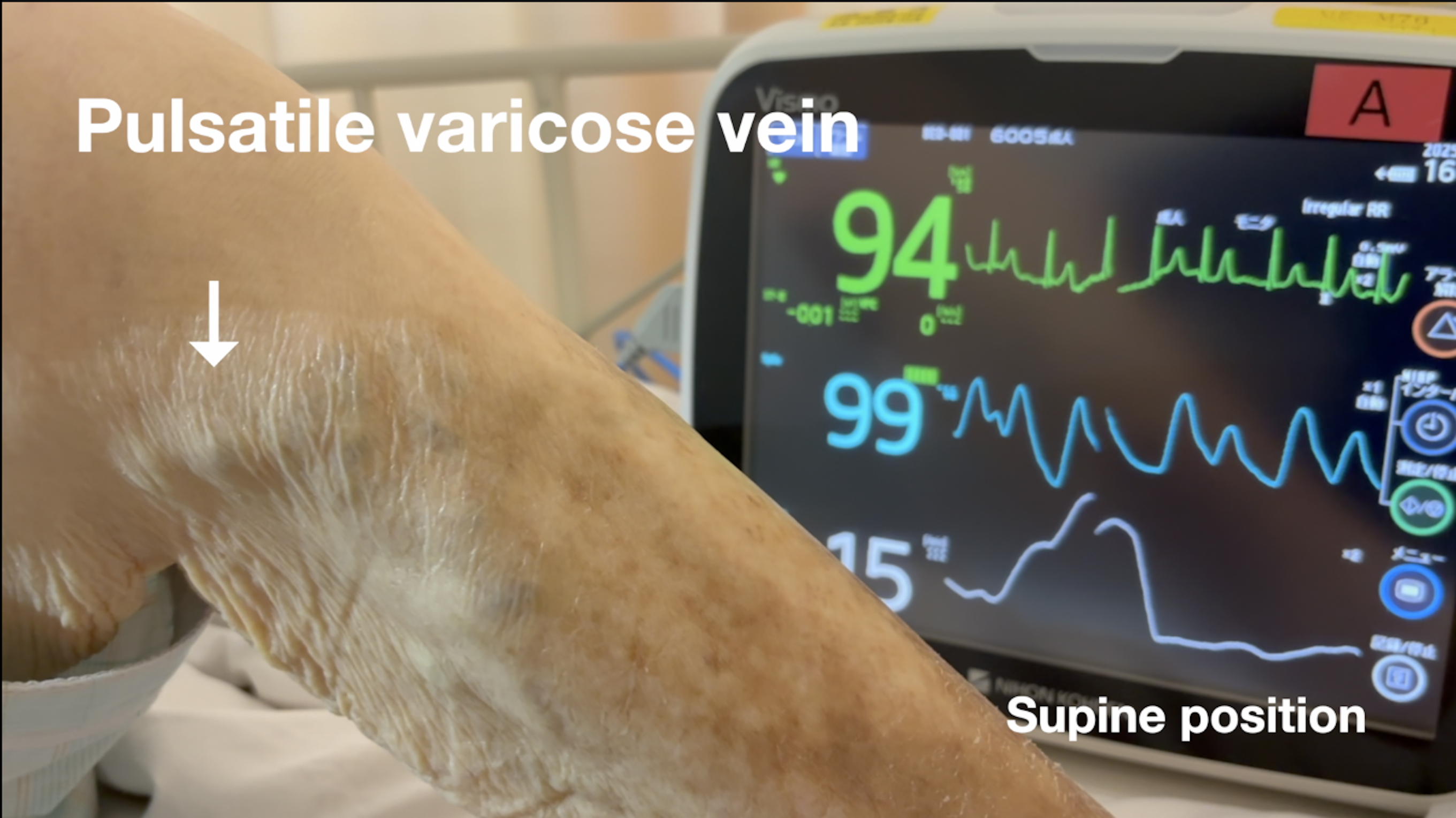

Supplement: Supplementary file 2 — Data S1: Supporting Information. [file CCR3-14-e72331-s001.tiff]
